# 41 miR-146a Mimic Administration Attenuates Lung Inflammation After Alcohol and Burn Injury

**DOI:** 10.1093/jbcr/irae036.041

**Published:** 2024-04-17

**Authors:** Connor O Guzior, Mashkoor A Choudhry, Carly Herrnreiter, Xiaoling Li, Abigail Cannon

**Affiliations:** Loyola University Chicago, Chicago, Illinois; Loyola University Chicago, Maywood, Illinois; Loyola University Chicago, Chicago, Illinois; Loyola University Chicago, Maywood, Illinois; Loyola University Chicago, Chicago, Illinois; Loyola University Chicago, Maywood, Illinois; Loyola University Chicago, Chicago, Illinois; Loyola University Chicago, Maywood, Illinois; Loyola University Chicago, Chicago, Illinois; Loyola University Chicago, Maywood, Illinois

## Abstract

**Introduction:**

Alcohol is a major confounding factor in pathology associated with burn injury. Patients intoxicated at the time of burn injury exhibit increased susceptibility to infection, increased risk of sepsis, multiple organ failure, and higher mortality rates. There are studies suggesting an association between excessive and prolonged lung inflammation and oxidative stress after burn injury, but the underlying pathophysiologic mechanisms are not fully understood. MicroRNAs are small noncoding RNA molecules that control gene expression by binding to mRNA and mediating repression of transcription or by promoting mRNA degradation. Recent findings from our laboratory have shown that in vivo administration of miR-146a mimic significantly reduced small intestine inflammation following alcohol and burn injury, characterized by reduced expression of IL-6 and reduced levels of neutrophil markers. In this study, we analyzed the expression of miR-146a and that of KC, Ly6g, IL-6, and MPO in lung tissue after in vivo administration of miR-146a mimic into mice to further demonstrate the role of miR-146a in lung inflammation following alcohol and burn injury.

**Methods:**

One day prior to alcohol and burn injury, male C57BL/6 mice were administered a miR-146a mimic or scramble via intraperitoneal injection. On the day of combined insult, each mouse received a single gavage of 0.4 mL 25% ethanol and four hours later they were given a ~12.5% total body surface area full thickness scald injury. One day following alcohol and burn injury, the mice were euthanized and lung tissue was harvested. Total RNA was extracted from the lung tissue and used for RT-qPCR analysis of miRNA and mRNA expression. Lung tissue homogenates were processed for ELISA analysis.

**Results:**

Following alcohol and burn injury, we found a significant decrease in miR-146a expression compared to sham vehicle (p < 0.05). While mice treated with miR-146a mimic showed an increase in miR-146a expression in lung tissue following ethanol burn injury, this was not found to be different from mice treated with scrambled control (p = 0.120). However, the treatment of ethanol burn mice with mimic significantly reduces the lung inflammation as assessed by IL-6, KC and Ly6g gene expression (p < 0.05) compared to those treated with scrambled control. MPO gene expression was also found to be reduced in ethanol burn mice treated with the miR-146a mimic, but it failed to reach significance (p = 0.171). Additionally, IL-6 protein levels trended downwards in mice after in vivo administration of miR-146a mimic following alcohol and burn injury, but they were not statistically different from ethanol burn mice treated with scrambled control (p = 0.488).

**Conclusions:**

Overall, these findings suggest a role for miR-146a in lung inflammation after alcohol and burn injury; more studies are undergoing to further confirm the role of miR-146a in lung inflammation and barrier integrity post-burn and alcohol injury.

**Applicability of Research to Practice:**

Above

